# Juggling School and Work From Home: Results From a Survey on German Families With School-Aged Children During the Early COVID-19 Lockdown

**DOI:** 10.3389/fpsyg.2021.734257

**Published:** 2022-01-31

**Authors:** Deborah Canales-Romero, Axinja Hachfeld

**Affiliations:** ^1^Empirical Educational Research, Department of History, Sociology, Empirical Educational Research and Sport Science, University of Konstanz, Konstanz, Germany; ^2^Pädagogische Hochschule Thurgau, Kreuzlingen, Switzerland

**Keywords:** COVID-19, distance-learning, homeoffice, stress, stressor, caregiver, parents, homeschooling

## Abstract

As consequence to the coronavirus outbreak, governments around the world imposed drastic mitigation measures such as nationwide lockdowns. These measures included the closures of schools, hence, putting parents into the position of juggling school and work from home. In the present study, we investigated the well-being of parents with school-aged children and its connection to mitigation measures with particular focus on parental roles “caregiver,” “worker,” and “assistant teacher” as stressors. In addition to direct effects, we expected indirect effects on well-being through changes in household dynamics. Data were collected via an online survey (*N* = 1313, 85.5% female; 53.2% university degree) conducted during the first wave of school closures in Germany. We observed that during the early COVID-19 pandemic, parental well-being in general was quite positive. Comparing the positive and negative PANAS subscales, parents agreed significantly more with the positive than with the negative items, *t*(1299) = 28.55, *p* < 0.001. Parents also reported an increase in positive family activities during the lockdown, *t*(1272) = 43.96, *p* < 0.001. Although a significant increase in negative household dynamics, such as disputes, was also observed to a lower extent, *t*(1295) = 7.78, *p* < 0.001. Using structural equation modeling, we observed that “homeoffice” was not significantly related to parents’ well-being, but positively affected household dynamics. Taking on the role of “assistant teacher” was negatively related to household dynamics. Additionally, we found a significant direct effect on negative affect for “assistant teacher.” We conclude that parents of school-aged children have mostly been able to establish positive dynamics in their households during the lockdown given the extra time they got to spend with their children by working from home. However, our results identify the role of “assistant teacher” as a potential stressor for parents. Bridging the gap between teachers and parents seems warranted especially if (some) distance-learning continues, in order to avoid long-term consequences for the students.

## Introduction

The coronavirus disease (COVID-19) outbreak turned into a worldwide crisis in the beginning of 2020. Although three major influenza epidemics (Kilbourne, 2006) and many other non-influenza ones---such as HIV---had been recorded in the 20th century, none had reached the levels of global propagation and fatality within such a short timeframe as the 2019 pandemic caused by SARS-CoV-2. Consequently, many governments decided on imposing drastic mitigation measures such as nationwide lockdowns, which included the closure of non-essential businesses, universities, child daycare centers and schools. After it was clear that in-person teaching would not resume for a considerable amount of time, schools around the world continued their curricula remotely via online platforms^1^. This meant not only that all workers from non-essential businesses should work from home (do ‘‘homeoffice’’^2^); but also, in case of parents, they had to simultaneously care for their children. For families with school-aged children, parents had to help them study from home and take on a role as “assistant teacher.” In essence, parents were burdened with juggling three concurrent roles from home: worker, parent, and assistant teacher.

Previous research on the effects of (short-term) school closures suggest they are considerably impactful measures against an outbreak (Ferguson et al., 2006). A study in the United States (Johnson et al., 2008) inquired about the hardship faced by families with school-aged children brought on by school closures due to an influenza B outbreak in North Carolina in late 2006. It found that parents’ attitudes toward the measure were overwhelmingly positive and the measure did not represent significant hardship for the affected families. However, the localized nature of the studied influenza outbreak differed in proportion and contagiousness to that of the COVID-19 outbreak. Dealing with this outbreak involves longer-term and more stringent mitigation measures—including not only school closures, but the halting of most activities that require close social contact. Thus, calling into question the applicability of the aforementioned study’s results for the current pandemic, and also about the generalizability of country-specific results due to diverse handling of the crisis by different governments. Some evidence to that effect was found in an early COVID-19 study in Canada (Findlay and Arim, 2020), which reported that anxiety levels were high regarding family stress during the lockdown. So although the empirical base about the psychological and social consequences of the pandemic is growing rapidly, its full impact—especially on families—is presently unknown (cp. Prime et al., 2020) and thus requires further study.

According to some, the COVID-19 pandemic and its associated mitigation measures will have similar effects to those of ecological disasters, terrorist attacks, political coups and other catastrophic events (Baker et al., 2020). It is important to know if the mitigation measures used around the world against COVID-19 are having adverse side effects on families because these can spill over within the family system and continue to be felt by the members and families as a whole in the years to come. Armed with this information, governments could improve such measures in future events of a similar nature or enact countermeasures. Due to the lack of information on the impact this type of crisis has on families with school-aged children, the focus of the present study was to investigate how families in Germany^3^ were coping and what effect the mitigation measures (especially, the closures of schools and non-essential businesses) have had on the well-being of parents in particular.

### The Family as a Dynamic System

The family is the most immediate context for the individual and has been described by social scientists as the primary social unit (Ebrahim and Ebrahim, 1982). Families perform multiple functions that provide benefits to individuals within them and to our society (Patterson, 2002). These functions are: (1) membership and family formation, (2) economic support, (3) nurturance, education, and socialization, (4) protection of vulnerable members.

The bioecological model by Bronfenbrenner and Morris (2006) highlights that the family is embedded in the community level, and the community level is, in turn, embedded in the social level. Because of their nested-ness, the different levels have mutual influence. This underlines the importance of studying families: because it provides a picture of an individual within his/her context. As a family is established through (commonly) the union of a couple, a set of habits, rituals and (tacit) rules are slowly forged by the members through their interaction (Ford, 1983; Fiese et al., 2002). At the same time that each of the members help forge this “family dynamic”, they are also influenced by it in a feedback loop (see Prime et al., 2020). It is the mechanism through which family members influence each other and their dyadic relationships. Children are especially susceptible to changes in rituals, habits and rules, possibly impacting their developmental outcomes (Browne et al., 2015). Therefore, any crisis or event that shifts these dynamic processes could have lasting spillover effects to be felt for many years to come.

This notion about how family members influence each other and their dynamic is the one of the basic tenets of systems theories (Prime et al., 2020). Parents, as the leaders of families, have a main role in shaping family dynamics. Thus, changes in their well-being can result in changes in the family system, also referred to as spillover or “cascading effects” (Prime et al., 2020). It is often helpful to compare the family system as a cog machine, so a change or disruption in one will in turn change or disrupt all of the others. So, any stressors affecting parental well-being negatively can also affect the well-being of the children negatively (Baker et al., 2001; Kaplan et al., 2001; Yotyodying and Wild, 2016). As an example, some of the “cascading effects” associated with lower levels of well-being amongst parents are harsher parenting practices and favoritism (Prime et al., 2020), which in turn have effects on the children’s well-being.

However, Prime et al. (2020) identify the family system also as a source for resilience because a well-functioning system can buffer negative effects of social changes on parents’ and children’s well-being. The defining feature of the family system are the relationships between its members which likely have changed due to the mitigating measures. With schools and workplaces closed, most families were spending more time together. While previous research shows that this can increase conflicts between all family members (Prime et al., 2020), it might also have positive effects if parents use the opportunity to spend more quality time with their children.

In sum, in this study, we investigate how the social changes, i.e., the mitigating measures, are related to parental well-being. The mitigating measures could affect parental well-being directly or indirectly, through changes in family or household dynamics. Families could differ with regard to how the mitigating measures affect their household dynamics. While we assume that the mitigating measures are stressors that are more likely to lead to negative changes, positive changes are also plausible. Hence, in this study, we were also interested in understanding how the family or household dynamics (namely, frequency and type of bonding activities and arguments) have changed since the introduction of COVID-19-related mitigation measures and how these changes relate to parental well-being.

### Effects of Mitigating Measures on Well-Being and Changes in Household Dynamics

In Hobfoll’s (1989) conservation of resources model, stress is an individual’s reaction to any type of loss: both perceived and actual loss, or even lack of gain. These situations that signify loss are also known as stressors. The direct threat of COVID-19 is the loss of health or potential loss of life of one or more members of the family. However, in the particular case of the COVID-19 pandemic, we can expect that this crisis will have also indirect effects on families and family members due to the mitigation measures imposed by governments (Brown et al., 2020). These mitigation measures have changed the quantity and quality of roles parents take on, thereby affecting parents well-being directly but also indirectly via changes in household dynamics. We will now discuss the two avenues of effects that we will focus on in this study.

#### Direct Effect on Well-Being

Stressors can have a direct psychological effect on individuals by the simple virtue of being. Hence, the mere knowledge of the pandemic and/or the mitigation measures themselves could already have adverse effects on individuals. Previous studies have established the direct link between external stressors and well-being (Errázuriz Arellano et al., 2012; Cobham et al., 2016), and recent studies during the COVID-19 pandemic have confirmed it (Masten and Motti-Stefanidi, 2020; Achterberg et al., 2021). Data from the German Socio-Economic Panel (SOEP), a household survey with approximately 15,000 households, showed a decline in life satisfaction in parents, as well as satisfaction with family-life during the pandemic. To test this, the researchers compared data from 2018 with data from May–June 2020. Parents with children below 11 years of age were especially prone to a decrease in levels of satisfaction (Huebener et al., 2020). The highest decline was found in parents with children under the age of three. Similarly, a longitudinal study during the pandemic among parents with preschool children found an increase in self-reported stress levels from November 2020 to beginning of 2021. Parents whose children had to stay at home due to the mitigating measures reported the highest level of stress (Autorengruppe Corona-KiTa-Studie, 2021).

Given the self-reinforcing nature of the family system and that parents are the family leaders—and are therefore the ones who putatively lead the family to manage the lockdown by adapting their habits, rituals and rules—we think it is particularly important to check for their well-being as a proxy or indicator of how the family is doing and will do in the future.

#### Changes in Household Dynamics Due to Changes in Parental Roles (Indirect Effect on Well-Being)

As we have mentioned before, parental roles have changed as consequence not of the pandemic, but of the mitigation measures enacted in every respective country. Here we go more in depth regarding the general changes brought on by the mitigation measures on the roles parents play in the household.

##### Caregiver

With the closure of all non-essential businesses, families have found themselves homebound with few entertainment and recreational options. Social-distancing required to avoid close contact people outside one’s own household circle. This is a considerable change to the everyday life of most families and will require for the family habits, rituals and rules to adapt to this new reality. The process of adapting to change involves the whole family, however, parents as the leaders of the unit will need to use more of their resources to lead the family in such process. It is expected that the normal levels of stress related to parenting (Greenberger and O’Neil, 1993) and family stress (Patterson, 2002) will be elevated by this adaptational process. We believe that due to these changes, the role of “caregiver” will pose more stress than usual, particularly if this role is unequally distributed among the parental dyad, i.e., one parent takes on more of the “caregiving” responsibilities than the other. For Germany, the main caregiver is usually the mother. This in mind, it is not surprising that Huebener et al. (2020) found gender differences in family life satisfaction (lower in mothers than in fathers) in families with small children during the COVID-19 lockdown in Germany, that the authors attributed to the higher share of caretaking responsibilities on the mothers’ side. However, for some families the extra time they have to spend together may provide an opportunity to engage in activities that promote the bonding and enrich dyadic relationships (Huebener et al., 2020). This has already been observed during the pandemic, as shown by a study from the United Kingdom (Benzeval et al., 2020). Cohen et al. (2020) also found evidence for positive as well as negative changes. While parents agreed to enjoy spending more time with their children and with the family, they also found it difficult to reconcile work and family. As a consequence, many parents agreed that they feel stressed because of the many obligations and challenges. Parents who reported having financial worries were significantly more likely to agree with items assessing negative household changes compared to parents without financial burdens.

##### Assistant Teacher

Although schools in most countries closed down, education was expected to continue – also referred to as emergency remote education (ERE; Letzel et al., 2020). While some countries were well-prepared to shift from classroom teaching to online teaching (because the infrastructure was already available), other countries struggled finding ways to implement it. For Germany, a country in which homeschooling is legally prohibited, the sudden change to distance-learning posed particular problems (for a distinction between “homeschooling” and “distance-learning” see Jolly and Matthews, 2018). In comparison to other Western countries, Germany lagged behind on digitalization, and the educational system in particular lacks the necessary structures for and experiences with digital teaching and learning (Bos et al., 2014; Eickelmann et al., 2019; Huber et al., 2020). Hence, before the pandemic teachers used digital media less often than teachers in other countries and often feel ill-prepared to handle it (Bos et al., 2014; Eickelmann et al., 2019). When schools first closed down, on March 16*^th^*, 2021, there was no system in place to give structured support and guidance to the teachers. Instead, schools were left alone with how to organize their teaching, which formats to implement (synchronous or asynchronous, etc.), which digital platforms to use and how and how often to get in contact with the students and their parents. It is not surprising then that during the first lockdown parents reported low levels of contact with the teachers (Porsch and Porsch, 2020; Wildemann and Hosenfeld, 2020; Steinmayr et al., 2021; Ulrich et al., in press) and a reduction of individual support (Wildemann and Hosenfeld, 2020). Instruction was often implemented by sending out tasks once a week and requiring students to send their answers back (Steinmayr et al., 2021). At the same time, feedback on the sent solutions was often not provided; and importantly, teachers were not allowed to grade student assignments during the first lockdown. In the study by Steinmayr et al. (2021), parents reported between 33 and 50% feedback rates from science/biology and language arts teachers, respectively. Video conferencing or other distance-learning activities were rarely held, and if so, would often take place only once a week (Steinmayr et al., 2021). The school closures, therefore, resulted in a loss of external structure that was not buffered by a new structure provided by the teachers. In light of this, and in comparison to regular classroom adherence, adherence to “homeschooling” is likely to be (more) dependent on (1) personality traits of the students themselves (cp. Martarelli et al., 2021), and (2) on the parents. An early German study reported that, indeed, parents were feeling overwhelmed by their new role as assistant teachers and stressed because of their inexperience with it (Letzel et al., 2020). These findings are echoed by another study done on parents with school-aged children in Poland, who found that educating their child at home was a “difficult” task (Parczewska, 2020). On that account, we chose to focus also on how this particular role as a “teaching assistant” would affect parents and the family as a whole.

##### Worker

As previously mentioned, parents who work have an additional role to juggle during this crisis, which is their role as a worker. For many, the work location shifted from the workplace to the “office at home.” This lack of physical separation between home and work could have effects in their productivity and motivation, which in turn might cascade into frictions in the dyadic parent-child relationships. In order to organize work and childcare, approximately half of the parents in a german study shifted their work time (e.g., worked early in the morning, late at night, or during weekends) or took turns (Autorengruppe Corona-KiTa-Studie, 2021). Yet, working at home could also have beneficial effects by making it easier to work and care for the children simultaneously, not having to waste time commuting or being in the office when there is not much work to do, thus allowing for parents to use this time differently. An early German study that focused on the impact of working from home during the COVID-19 crisis had on workers who were not used to it, found that workers seemed to be adapting well to the situation and their well-being improved during the 2-week study period (Schade et al., 2021). However, this study did not particularly focus on parents. For those whose work could not shift to the homeoffice, working hours were often reduced by the employers (“short-time work”), and employees received government-funded “short-time work money.” Möhring et al. (2021) found that having to reduce their work time did in fact negatively affect mothers’ well-being, while it did not affect fathers’.

**FIGURE 1:**
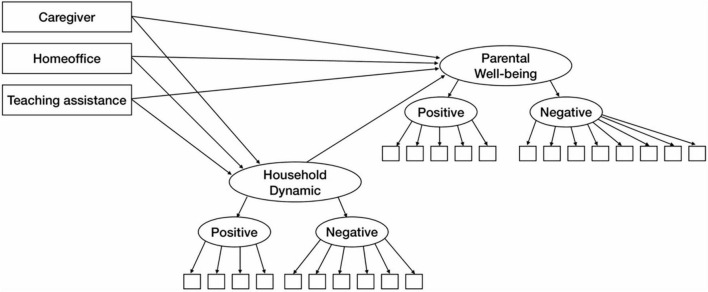
Hypothesized model for partially latent structural regression.

Taken together, we hypothesized that during the enactment of mitigation measures against COVID-19, the changes in these three roles (“caregiver,” “teaching assistant,” and “worker”) will result in a change in family dynamics within the household. In line with the transactional theory of stress and the conceptional framework on family functioning proposed by Prime et al. (2020), we assume that there is a direct relation between parental well-being and these stressors; and also an indirect effect via the changes in household dynamics (see Figure 1). Since the changes in household dynamics could be negative and positive, we consider both aspects in our study.

In our study, we focus on parental well-being during the first lockdown in German families and investigate two questions:

(1) How was parental well-being affected by the mitigating measures which led to changes in the three roles of caregiver, worker, and assistant teacher?
(2) Can part of the effect of these stressors on well-being be explained by changes in household dynamics?

## Materials and Methods

### Procedure and Participants

For this study, an online survey was programmed in German and hosted on the SoSci Survey platform^4^. Parents were invited to answer the 15-min questionnaire. At the beginning of the questionnaire, participants gave informed consent or else were redirected to an exit page. Convenience sampling through multiple-medium promotion (exclusively online, e.g., via Facebook, Twitter, e-mails to parent representatives) yielded 1,725 participants mostly from the German state of Baden-Württemberg (76.6%). Cases in which more than 50% of the questionnaire data was missing were excluded from analysis, resulting in 1,313 cases to be analyzed (85.5% female; 53.2% with university degree, further details in Table 1). The excluded cases were exclusively due to participant dropout before answering 50% of the questions, as it was deemed that we could not rely on participant seriousness when this threshold had not been reached. Data were gathered in April 2020 during the ‘‘first wave’’ of school lockdowns, more precisely between the third to fifth week after government-mandated school closures in Germany.^5^ We aimed at this early period to catch the time of greatest instability. We assumed that during the first weeks, parents and children would not yet have had enough time to adapt to the change and develop new daily routines.

**TABLE 1:** Sample descriptive statistics.

Variables	*n*	%	*n*	%	*n*	%
**Child**	**Total sample**					

Year of birth	1232					
2007–2010 (10–13 years old)	720	54.9%				
Sex	1301					
Female	646	49.2%				
Male	649	49.4%				
Diverse	6	0.5%				
Disability	1303					
Yes	67	5.1%				
No	1236	94.1%				

**Parent**	**Total sample**		**Fathers**		**Mothers**	

Teacher	1256	–	129	–	1120	–
Yes	153	11.6%	16	12.4%	135	87.7%
No	1103	84.0%	113	87.6%	985	12.0%
Education	1257	–	129	–	1123	–
None	3	0.2%	1	0.8%	1	0.1%
Volkschule/Hauptschule	35	2.7%	3	2.3%	32	2.8%
Realschulabchluss/Mittlere Reife	271	20.6%	24	18.6%	246	21.9%
Hochschulreife/Abitur	249	19%	16	12.4%	232	20.7%
Hochschulabschluss	699	53.2%	85	65.9%	612	54.5%
Relationship to child	1253	–	–	–	–	–
Father figure	129	9.8%	–	–	–	–
Mother figure	1123	85.5%	–	–	–	–
Other	1	0.08%	–	–	–	–
Single parent	1253	–	129	–	1123	–
Yes	139	10.6%	6	4.6%	133	11.8%
No	1030	78.4%	113	87.6%	916	81.6%
Partly/mostly	84	6.4%	10	7.7%	74	6.6%
Main caregiver (of child/children)	1024	–	113	–	910	–
Myself	569	43.3%	10	7.7%	559	49.7%
Both of us together	403	30.7%	71	55.0%	332	29.6%
Partner	47	3.6%	30	23.3%	16	1.4%
Other person	5	0.4%	2	1.5%	3	0.3%
Work status	1241	–	126	–	1085	–
Retired	9	0.7%	3	2.3%	6	0.5%
Looking for a job/unemployed	59	4.5%	3	2.3%	56	5.0%
Parental leave	38	2.9%	0	0.0%	9	3.4%
Studying	21	1.6%	1	0.8%	20	1.8%
Mini job	39	3.0%	0	0.0%	39	3.5%
Part-time job	621	47.3%	18	13.9%	603	53.7%
Full-time job	304	23.2%	84	65.1%	220	19.6%
Freelance/company owner	150	11.4%	17	13.2%	132	11.8%
Income rating	1251	–	128	–	1121	–
Live comfortably	658	50.1%	64	49.6%	594	52.9%
Get by	499	38.0%	55	42.6%	444	39.5%
Difficulty getting by	68	5.2%	7	5.4%	61	5.4%
Only barely get by	26	2%	2	1.5%	22	1.9%
Homeoffice (those who work)	1122	–	121	–	1000	–
Yes	546	41.6%	75	58.1%	471	41.9%
Partly	188	14.3%	17	13.2%	170	15.1%
No	388	29.6%	29	22.5%	359	32.0%
Assistant teacher	1286	–	128	–	1114	–
Strongly disagree	236	18.0%	26	20.1%	199	17.7%
Disagree	406	30.9%	34	26.4%	364	32.4%
Agree	437	33.3%	51	39.5%	368	32.8%
Strongly agree	207	15.8%	17	13.2%	183	16.3%

*Missing data suppressed from this table but considered for percentages. N for each category title represents the amount of people who answered the item(s) from the total sample used. For brevity, only the most represented category of year of birth is reported here. Further breakdown of parent descriptives according to parent gender (only fathers and mothers) included. Parent gender was assessed via the question “relationship to the child,” in which the category of “other” and no answer was possible.*

Interested participants could join a lottery of 20 gift cards with a value of 25€ each. For the study design and procedure, established ethical guidelines for psychological research were followed.

Although parents with children of any age were allowed to participate, we encouraged them to focus on a single child—preferably the one closest to the age of 12, i.e., the 6th grade in the German educational system—to answer the questions. This focus was of particular interest for the researchers for two reasons: first, most children change from elementary school to secondary school at around 10 or 11 years of age (after fourth grade) and this change puts academic demands on the children; hence, we expect families to be still concerned about the educational progress of their child at this stage. Secondly, children at the age of 12 are already expected to work independently on their school tasks even during distance learning; therefore, we expected parents of children that age to be fluctuating between providing support and expecting independence. In our sample, most children (55.24%) were in secondary school, which comprises 5th to 10th grade in most of Germany (except Berlin were children change after grade 6), and of these children, the majority (64.83%) were in the highest (academic) track secondary school (“Gymnasium”).

Regarding parent and family characteristics, the sample resembles that of other studies conducted during the school lockdown (e.g., Porsch and Porsch, 2020; Steinmayr et al., 2021). Families with higher educational degrees and higher socio-economic background were overrepresented (Table 1). We asked participants how they get along economically and 88% answered to get by well or very well. Roughly, 85% percent said they had enough space in their current living situation. For our study, we asked families to let the parent who is mainly responsible for childcare and distance teaching answer the questionnaire. Thusly, most respondents in our study were female (85.5%)—as in other corona studies—suggesting that mothers are over-proportionally responsible for the children during the pandemic. Further sociodemographic statistics are summarized in Table 1.

### Measures

The parent survey assessed a variety of questions capturing how families had experienced and coped during the lockdown, and how they had organized their family life and distance learning. In the present analyses, we focus on parental well-being as our main outcome of interest. Following the conceptional framework on family functioning proposed by Prime et al. (2020) and the transactional theory of stress, we understand the social changes, represented by the mitigating measures, as stressors that influence parent’s well-being and family’s household dynamics directly. Changes in household dynamics, in turn, could either amplify negative effects of the mitigating measures or—when changes are positive—serve as a buffer and a source of resilience.

#### Well-Being

In order to assess parents’ well-being, we used an abbreviated version of the Positive and Negative Affect Schedule (PANAS; Krohne et al., 1996). Usually consisting of 20 items, we selected the 12 most relevant items for the lockdown situation: five items for positive affect and seven for negative affect (all questions used for this study can be found in Supplementary Appendix A). Because of the uncertainty of the unprecedented situation, we also included the additional item “unsettled” (original: “verunsichert”), resulting in 13 items. The traditional 5-point Likert response scale was used, ranging from 1 (“very slightly or not at all”) to 5 (“extremely”). The instruction was modified to “How have you felt in the last days [since the lockdown]?” in order to only capture the current emotional state of the individuals since the lockdown and not their general feeling (trait). To avoid priming effects, the PANAS was presented before the other scales, but after asking their children’s demographics. Our abbreviated version of the PANAS scale had a good internal consistency for positive (Cronbach’s α = 0.74) and negative items (Cronbach’s α = 0.85) despite the fewer number of items in the former.

#### Household Dynamic

Parents were asked a series of 10 items related to the household activities/events during the school closures compared to their frequency before the school closures. We included positive and negative household activities/events. The positive items focused on activities that families could do together at home despite the lockdown measures and that could be described as “quality time.” Examples for positive activities or events are “cooking or eating together” or “doing recreational activities together like playing board games or music together.” The negative items focused (1) on the relationship between parent and child or between the siblings, since previous research has shown that similar threats can increase the potential for conflicts, arguments, and aggression. Example items are: “dispute about use of smartphones, tablets or similar” or “dispute with siblings.” Also, because we were interested in the effects of the school closures, negative items (2) focused on schoolwork related negative events. Examples are: “dispute about completing schoolwork” or “dispute about checking the schoolwork.” Answers were given on a scale from 1 (“much less often”) to 5 (“much more often”), with 3 being a theoretical middle point of “same/no change.” The only exception to this was the following item: “My child reacts annoyed when I explain schoolwork to him/her,” which only had a response range from 1 (“strongly disagree”) to 4 (“strongly agree”). To be included in the scale, the response range was standardized to match the others. The full scale included four positive (Cronbach’s α = 0.62) and six negative items (Cronbach’s α = 0.79), which showed acceptable internal reliability. Although the positive scale’s reliability is considerably lower than the negative ones, this might be due to a lower number of items. However, this can still be considered an acceptable reliability value for psychological constructs (Field, 2013).

#### Stressors

To assess changes in the family’s lifestyle that the pandemic has brought on, three single items were included. These three items aimed at three central stressors brought on by the mitigation measures on families with children that we wanted to focus on, namely on taking on the role of “assistant teacher,” doing “homeoffice,” and “caregiver.” To assess requirements of distance learning, the respondents were asked the following question: “The teachers of my child include me to provide learning support,” with answers from 1 (“strongly disagree”) to 4 (“strongly agree”). To assess the working conditions, we added the following item: “Can or must you currently work from home (because of the Corona protection regulations)?”, with answers from 1 (“yes”), 2 (“in part”) and 3 (“no”). To assess amount of childcare responsibility, we asked: “Who is in your home the main person in charge of taking care of the child?”, with answers from 1 (“myself”), 2 (“my partner”), 3 (“both of us together”), and 4 [“other person(s)”]. This variable was dummy coded into involved in child rearing or not, with answers “my partner” and “other person(s)” coded 1, and “myself” along with “both of us together” coded 2.

### Data Analysis

Data were analyzed using RStudio (Team R Studio, 2020) version 1.3.1073 “Giant Goldenrod.” In order to test the interrelation of all of our variables in context, we decided to use structural equation modeling (SEM) using the *lavaan* package version 0.6-7. Zero-order correlations were obtained using the *rcorr* function of the *Hmisc* library. Figure 1 depicts our hypothesized model and how we considered our variables to be interrelated. For the final model, we controlled for socioeconomic status.

Missing data was treated using pairwise deletion for the descriptive statistics and zero-order correlations. Due to the limited nature of the response scales (1–5), outlier deletion was not deemed necessary. The total number of used observations in the SEM model was *n* = 960 (only complete observations used); using Full Information Maximum Likelihood (FIML), that number increased to *n* = 1301. The Goodness of Fit Indices (GFI) cutoff values used were those compiled by Kline (2005) for SEM models: non-significant χ^2^, CFI > 0.9 as “good”, RMSEA < 0.10 as “acceptable” and <0.08 as “good,” SRMR < 0.08 as “good.” On account of the large sample size, the chi-square value was not considered to assess the fitness of the model, yet is still reported. For the measurement part of the model, a cutoff value of 0.40 for factor loadings was considered, as low factor loadings could indicate that the latent variable is not adequately measured by that item and therefore should probably be discarded (Chin, 1998; Hair et al., 2011).

## Results

### Descriptive Results

To explore how parents experienced the first lockdown emotionally, we analyzed the PANAS data (Table 2). Comparing the positive and negative affect subscales, the positive items received significantly higher scores (*M*_*PA*_ = 2.92, *SD*_*PA*_ = 0.68) than the negative items (*M*_*NA*_ = 2.06, *SD*_*NA*_ = 0.72), *t*(1299) = 28.55, *p* < 0.001, suggesting that parents were experiencing more positive than negative emotions during the time of the lockdown. The positive item with a highest score was “attentive” (*M* = 3.50, *SD* = 0.88). Whereas the negative item with the highest score was “distressed” (*M* = 2.51, *SD* = 1.04). The item we added to the modified PANAS scale for this particular study (“unsettled”) was within the range of the rest of the negative items (*M* = 2.27, *SD* = 1.03). Fathers and mothers did not significantly differ in average scores for the positive [mothers: *M* = 2.92, *SD* = 0.67; fathers: *M* = 2.94, *SD* = 0.67; *t*(158.67) = 0.31, *p* = 0.75] or the negative scale [mothers: *M* = 2.06, *SD* = 0.71; fathers: *M* = 2.01, *SD* = 0.74; *t*(156.29) = −0.75, *p* = 0.45].

**TABLE 2:** Itemized response percentages for the PANAS scale, grouped by subscale.

	Positive scale%						Descriptives	
Item	1	2	3	4	5		*M*	*SD*
1 Active	3.0	14.1	45.7	28.1	8.0		3.24	0.90
6 Inspired	19.1	31.2	34.3	12.6	1.5		2.45	0.99
8 Enthusiastic	28.7	27.8	30.0	10.2	2.3		2.29	1.06
10 Determined	6.9	17.4	39.6	28.0	6.7		3.10	1.00
11 Attentive	2.0	9.0	36.6	39.8	11.1		3.50	0.88
						Subscale	2.92	0.68

	**Negative scale%**							

2 Distressed	15.6	39.3	25.4	15.1	3.5		2.51	1.04
3 Upset	33.2	35.6	16.5	10.7	3.0		2.14	1.09
4 Startled	47.3	29.1	13.7	7.1	1.8		1.86	1.02
5 Hostile	73.6	16.1	6.5	2.1	0.4		1.37	0.74
7 Irritable	22.2	41.2	17.1	14.9	3.4		2.35	1.09
9 Nervous	39.2	31.7	15.2	11.3	1.4		2.03	1.06
12 Scared	40.8	37.7	11.2	7.3	1.8		1.90	0.99
13 Unsettled	22.0	45.5	16.2	12.0	3.0		2.27	1.03
						Subscale	2.06	0.72

*Response options 1 (“very slightly or not at all”), 2 (“a little”), 3 (“moderately”), 4 (“quite a bit”), 5 (“extremely”).*

Regarding the current household dynamics, we looked at the responses to the HHD scale (Table 3). Keeping in mind that this scale was designed to show relative frequency, an answer of 3 would represent no change in the frequency of activities relative to the time before the lockdown. Participants responded that the frequency of the inquired activities in the positive subscale was slightly, though significantly, higher (*M*_*P*_ = 3.65, *SD*_*P*_ = 0.54) during the lockdown than before, *t*(1272) = 43.96, *p* < 0.001. These activities included cooking or eating together (64.5% reported a higher frequency than before) or doing recreational activities together (57.7% reported a higher frequency than before). The change reported by parents for negative items was lower (*M*_*N*_ = 3.15, *SD*_*N*_ = 0.71), but still significantly different to the hypothetical midpoint of 3, *t*(1295) = 7.78, *p* < 0.001. For example, 34.6% reported more disputes about homework than before. Roughly half (50.2%) of parents reported that their children have been irritated when parents have to explain schoolwork. Similar to the PANAS scales, female and male caregivers did not significantly differ in average scores for the positive [mothers: *M* = 3.67, *SD* = 0.53; fathers: *M* = 3.64, *SD* = 0.56; *t*(155.92) = −0.60, *p* = 0.54] or the negative HHD scale, [mothers: *M* = 3.16, *SD* = 0.70; fathers: *M* = 3.09, *SD* = 0.69; *t*(159.52) = −1.01, *p* = 0.31]. Altogether, the HHD scale indicates that the household dynamic and atmosphere reported by the majority of parents in our sample has changed, with an increase in positive activities (especially cooking and sharing meals) compared to before the lockdown, and a lower but still significant increase in negative activities or events, like arguing.

**TABLE 3:** Itemized response percentages for the HHD scale, grouped by subscale.

Item content	Positive items		
	*1–2*	*3*	*4–5*
1. Cooking or eating together	1.3	30.6	64.5
2. Doing recreational activities together	6.5	31.7	57.7
3. Watch television together	5.7	46.9	40.8
8. Long conversations about a topic	2.3	38.1	56.1

	**Negative Items**		

4. Disputes about doing homework	14.9	42.6	34.6
5. Disputes about inspecting homework	14.9	48.9	24.3
6. Disputes about the use of electronic devices	9.8	46.3	35
7. Disputes about other topics	17.4	57.0	19.1
9. Disputes with siblings	12.6	36.6	30.9
10. My child reacts irritated when I explain schoolwork to him/her	48	–	50.2

*These are all percentages from the final sample used for analysis. Missings not shown in table but considered for percentages. Item 10 had a 1–4 scale, which was later standardized for analysis, hence the lack of observations in the middle value. Response options for items 1 to 9: 1 (“much less often”), 2 (“less often”), 3 (“same/no change”), 4 (“more often”), 5 (“much more often”).*

*Response options for item 10: 1 (“strongly disagree”) to 4 (“strongly agree”).*

### Structural Equation Modeling Results

As a first step, we ran correlations (Table 4) between the outcome variables of ‘‘parental well-being’’ and ‘‘household dynamic’’ with stressors brought on by the pandemic: ‘‘homeoffice,’’ ‘‘caregiver,’’ and ‘‘teaching assistant.’’^6^ Table 4 shows that having to assist in school teaching (“teaching assistance”) had a significant positive correlation with the negative subscales of both the PANAS, *r* = 0.17, *p* < 0.001, and the HHD scales, *r* = 0.24, *p* < 0.001. The amount of responsibility with the children (“caregiver”) was not significantly correlated with any of the outcome variables. The “homeoffice” variable showed a significant relationship with the positive subscale of the HHD, *r* = 0.19, *p* < 0.001, and a significant negative relationship with “caregiver,” *r* = −0.07, *p* < 0.025.

**TABLE 4:** Zero-order correlations, Cronbach’s alphas, means, and standard deviations for main variables in this study.

	1	2	3	4	5	6	7
(1) PA_pos	(0.74)						
(2) PA_neg	−0.22***	(0.85)					
(3) HHD_pos	0.15***	−0.07*	(0.62)				
(4) HHD_neg	−0.24***	0.40***	−0.10***	(0.79)			
(5) Caregiver^†^	0.00	−0.01	−0.01	0.00	1.00		
(6) Homeoffice	0.04	0.00	0.19***	−0.01	−0.07*	1.00	
(7) Teaching assistance	0.00	0.17***	0.03	0.24***	0.02	0.03	1.00
*M*	2.92	2.06	3.65	3.1	1.95	2.14	2.48
*SD*	0.68	0.72	0.54	0.67	0.20	0.90	0.97
Scale	1–5	1–5	1–5	1–5	1–2	1–3	1–4

Higher scores on the last three variables indicate higher levels of responsibility in child rearing, having to do more homeoffice and more activities regarding schoolwork support. Cronbach’s alphas for each subscale are reported in parenthesis on the main diagonal where appropriate. PANAS positive subscale (PA_pos), PANAS negative subscale (PA_neg), HHD positive subscale (HHD_pos), HHD negative subscale (HHD_neg). *p < 0.05, ***p < 0.001, ^†^biserial correlations.

Next, we followed the two-step approach to structural regression models by Anderson and Gerbing (1988), which suggests to first test the fit of the measurement part of the model and then add the structural part of the model. Both measurement models—HHD and PANAS—were tested first using the CFA (confirmatory factor analysis) function of *lavaan*. Improvements in fit were reached by adding correlated errors in the PANAS scale among items 12 (“scared”) and 13 (“unsettled”); as well as between items 3 (“upset”), 5 (“hostile”) and 7 (“irritable”). The correlated errors in the PANAS scale were well-justified as the word groups have very similar meanings; items 3, 5, and 7 are more related to anger whereas the other two items are more related to anxiety. These types of item groups with the PANAS scale have also arisen in the original version (Thompson, 2007). A three-factor model for the PANAS—to separate the anger-related items and the anxiety-related items—was attempted and did not result in a good fit (Table 5). The data suggested that a two-factor model was the best fit, χ^2^(60) = 451.3, *p* < 0.001, CFI = 0.934, RMSEA = 0.071, SRMR = 0.055.

**TABLE 5:** Attempted models and GFIs.

	χ ^2^	df	χ ^2^Δ (df)	CFI	RMSEA	CI	SRMR	AIC
**PANAS (measurement model)**								
Model A (two-factor model)	1,305.92	64	–	0.789^†^	0.123	0.117–0.128	0.081	42,474.79
Model B (three-factor model)	590	62	715.49(2)***	0.910	0.081^†^	0.075–0.087	0.060	41,763.30
Model C (two-factor model with correlated errors)	451.38	60	139.06(2)***	0.934	0.071	0.065–0.077	0.055	41,628.24

**HDD (measurement model)**								
Model D (two-factor model)	245.85	34	–	0.904	0.084^†^	0.074–0.094	0.058	20,863.11
Model E (two-factor model without items 7 and 9)	43.76	19	–	0.988	0.035	0.021–0.049	0.033	20,048.19

**PANAS and HDD (measurement model)**								
Model F	722.92	179	–	0.926	0.053	0.049–0.058	0.062	54,035.85

**Final model (measurement and structural parts)**								
Model G	786.14	226	–	0.915	0.051	0.047–0.055	0.056	46,368.89

**Final model with FIML**								
Model H	974.97	226	–	0.914	0.050	0.047–0.054	0.053	70,942.52

All χ^2^ values in this table are significant. Chi-square difference test values provided only for comparison among nested models. ***p < 0.001, ^†^beyond cutoff value.

Moving on to the HDD scale, items 7 (“disputes over other topics”) and 9 (“disputes with siblings”) were eliminated due to factor loadings below our selected threshold, yielding a model with a very good fit, χ^2^(19) 43.76, *p* = 0.001, CFI = 0.988, RMSEA = 0.035, SRMR = 0.033. A model including both measurement models also resulted in a good fit, χ^2^(179) = 722.92, *p* < 0.001, CFI = 0.926, RMSEA = 0.053, SRMR = 0.062. A second-order CFA was considered for both scales to better approximate the hypothesized model, however low correlation among the PANAS subscales (*r* = −0.26, *p* < 0.001) and HDD subscales (*r* = −0.18, *p* < 0.001) indicated the subscales were best represented as two different factors, not as parts of higher-order latent factors. Thus, we modified our hypothesized model accordingly.

**FIGURE 2:**
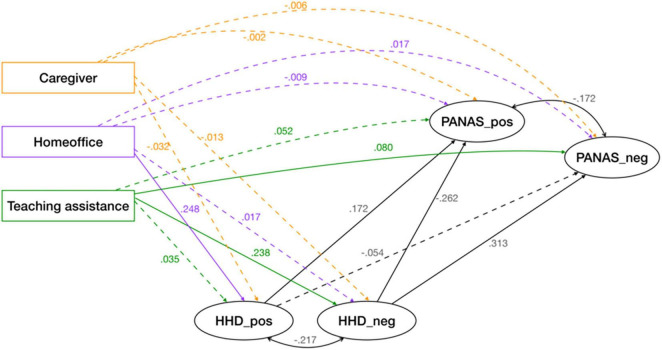
Final (structural) model for partially latent structural regression using FIML with standardized coefficients. Non-significant paths are in dotted lines, whereas significant ones are in solid lines.

Finally, we ran the analysis including the structural part of the model with possible mediation of our three selected stressors (“caregiver,” “homeoffice,” and “teaching assistant”) through household dynamics, including socioeconomic status as a control variable. The resulting model showed adequate global fit indices, χ^2^(210) = 786.814, *p* < 0.001, CFI = 0.915; RMSEA = 0.051, SRMR = 0.056. In order to ensure the robustness of the model, we also ran the model using FIML to treat missing data, which now used 1,301 cases and showed minimal changes in GFIs and estimates compared to the model without FIML, χ^2^(230) = 974.97, *p* < 0.001, CFI = 0.914; RMSEA = 0.050, SRMR = 0.053 (see Table 5 for a summary of models attempted and their GFIs). In this last model (Figure 2), the range of factor loadings for each of our latent factors was between 0.51 and 0.74 for the positive PANAS subscale, 0.44–0.76 for the negative, 0.45–0.73 for the positive HDD subscale and 0.49–0.90 for the negative.

Regarding how all endogenous variables are interrelated, we found parents’ well-being was directly related to the household dynamics (HHD) where positive HDD had a direct effect on positive emotions (β = 0.17, *SE* = 0.04, *p* < 0.001) and negative HDD a direct effect on negative emotions (β = 0.31, *SE* = 0.03, *p* < 0.001). Negative HDD were also related to fewer positive emotions (β = −0.26, *SE* = 0.04, *p* < 0.001), and positive HDD also related to fewer negative emotions, however, the latter relationship was not significant at the 0.05 level (β = −0.05, *SE* = 0.04, *p* = 0.17).

We were also interested in investigating whether the stressors (“caregiver,” “homeoffice,” and “teaching assistance”) directly or indirectly through HDD affected participants’ well-being. Analyzing the three stressor variables on the final model (Table 6), “caregiver” had no significant effect on any of the endogenous variables.

**TABLE 6:** Effects breakdown between exogenous and endogenous variables of the final model showing mediated effects.

	Endogenous variables							
	HHD_pos		HHD_neg		PA_pos		PA_neg	
Causal variables	β	*SE*	β	*SE*	β	*SE*	β	*SE*
**Caregiver**								
Direct effect	−0.032	0.034	−0.013	0.029	−0.002	0.032.	−0.006	0.030
Total indirect effect	–	–	–	–	−0.002	0.011	−0.002	0.010
Total effect	−0.032	0.034	−0.013	0.029	−0.004	0.033	0.009	0.031
**Homeoffice**								
Direct effect	0.248***	0.036	0.017	0.031	−0.009	0.036	0.017	0.034
Total indirect effect	–	–	–	–	0.038*	0.015	−0.008	0.014
Total effect	0.248***	0.036	0.017	0.031	0.029	0.035	0.009	0.034
**Teaching assistance**								
Direct effect	0.035	0.034	0.238***	0.029	0.052	0.032	0.080**	0.030
Total indirect effect	–	–	–	–	−0.056***	0.014	0.073***	0.013
Total effect	0.035	0.034	0.238***	0.029	−0.005	0.032	0.152***	0.030
**HHD_pos**								
Direct effect	–	–	–	–	0.172***	0.041	−0.054	0.039
Total indirect effect	–	–	–	–	–	–	–	–
Total effect	–	–	–	–	0.172***	0.041	−0.054	0.039
**HHD_neg**								
Direct effect	–	–	–	–	−0.262***	0.035	0.312***	0.033
Total indirect effect	–	–	–	–	–	–	–	–
Total effect	–	–	–	–	−0.262***	0.035	0.312***	0.033

Total indirect effects through different mediators (the two HDD subscales) were calculated by simple addition due to low correlation among HHD subscales. *p < 0.05, **p < 0.01, ***p < 0.001.

“Homeoffice” did not have a significant direct effect on the positive or negative affect; nevertheless, it did have a direct effect on positive HDD (β = 0.25, *SE* = 0.36, *p* < 0.001). Hence, results suggest an indirect (mediated) effect between homeoffice and well-being via positive HHD of 0.043 (0.25*0.17). However, its total effect on the positive PANAS is diminished (to 0.38) by the alternate indirect effect it has through its non-significant relationship via negative HHD, which subtracts 0.004 (0.017*–0.26). In the end, homeoffice has no significant total effect on well-being (Table 6).

‘Teaching assistance” was found to have a significant direct effect only on the negative PANAS subscale (β = 0.08, *SE* = 0.03, *p* = 0.009); and a positive direct effect on the negative subscale of the HDD (β = 0.24, *SE* = 0.03, *p* < 0.001). Consequently, it also had an indirect effect (partial mediation) on the negative PANAS subscale through the negative HDD subscale (as a reminder, β = 0.31, *SE* = 0.04, *p* < 0.001). Therefore, the indirect effects of “teaching assistance” on the negative PANAS subscale by way of the negative HHD amounts to 0.07 (0.24*0.31). In total, indirect and direct effects, “teaching assistance” has an effect on the negative PANAS of 0.15 (0.08 + 0.07), *R^2^* = 0.131, indicating that for every standard deviation increase in teaching assistance there is an increase of 0.15 standard deviations in the negative PANAS subscale. The variance in the negative PANAS explained by the model is 13.1%. Full mediation also occurs between teaching assistance and the positive PANAS because of significant indirect effects (β = −0.05, *SE* = 0.01, *p* < 0.001). However, the total effect of teaching assistance on positive affect is non-significant. The variance of the positive PANAS explained by the model is of 11.3%.

## Discussion

Our aims with this study were to investigate the well-being of parents with school-aged children, and how it was affected by mitigation measures imposed by the German government due to COVID-19 with particular focus on parental roles “caregiver,” “worker,” and “assistant teacher” as stressors. We also assumed that the stressors, i.e., the mitigating measures, would not only directly affect parental well-being but also indirectly by consequent changes in household dynamics. With this purpose we did a cross-sectional online survey in which we asked one parent per household to respond, preferably, the main caregiver of the children.

In our study, and contrary to our assumptions, participants seemed to experience the first wave of the lockdown rather positively—at least regarding to their well-being—notwithstanding the radical changes in everyday life that the mitigation measures entailed. The feelings they most endorsed were “active,” “attentive,” and “determined”. This seems to suggest that they might be aware of the challenges posed by the pandemic and are therefore engaging all their resources to handle it. Although we expected parents to feel “unsettled”—hence, adding this additional item to the original PANAS scale to accommodate for the unique situation of the pandemic—they did not report high levels of this affect during early lockdown. This might have been due (at least in part) to an increase in positive household dynamics. In our sample, positive activities in households increased more than negative ones (although these also increased to a lesser extent). Our SEM model results support the findings from the United Kingdom study (Benzeval et al., 2020) that doing more homeoffice has granted families the opportunity to do more positive activities together, such as cooking, eating and doing recreational activities; which in turn has a positive correlation with parents’ emotional well-being. We confirm the findings of Schade et al. (2021) that showed that German workers seem to be adapting well to the situation and reporting an increase in well-being during early lockdown. Different results were found by Möhring et al. (2021) who reported a decrease in well-being among mothers with pandemic-induced “short-work” schedules. This difference might be explained by financial concerns that go hand in hand with short-time work. The mothers in our sample were mainly able to work from home, hence, less likely to experience great financial losses. Furthermore, we did not find gender differences in the PANAS or the HHD scales in our sample. One reason might be the selection of the sample: we asked respondents that the person providing the answers to our survey should be the main caregiver of the children, hence, all the men included in the sample were more likely to take on the role that is usually taken by the mother.

On the other hand, there does seem to be issues with the change in dynamics regarding schoolwork. Our findings suggest that this new parental role of “assistant teacher” showed a direct positive relation with negative affect, and also a positive relation with the amounts of arguments and disagreements among parents and their children. According to our model, this last effect in turn, has a negative relationship on the well-being of parents via a twofold mechanism: increasing negative affect and reducing positive affect. This contrasts with the effects of homeoffice, which only had a direct positive relationship on positive household activities, thus increasing positive affect. In line with what Letzel et al. (2020) reported in their paper, we found that German parents were having difficulties with taking on the role of “assistant teacher” (for insightful highlights from parents responses to their qualitative interviews, see Letzel et al., 2020). In their study, Letzel et al. (2020) found a significant decrease in parental well-being during the early COVID-19 lockdown, whereas parents in our sample reported high levels of well-being. Yet, due the cross-sectional nature of our study, we cannot draw conclusion about changes in relative well-being. Another point of discrepancy is the use of a different well-being measure. We used a (modified) PANAS scale, as did Schade et al. (2021), whereas Letzel et al. (2020) used the Positive and Negative Activation and Valence (PANAVA) instrument. Another possibility to explain this discrepancy, depending on the order of the questionnaire items in the study by Letzel et al. (2020) are priming effects. Because parents are having negative experiences with distance-learning, this might have impacted their responses in the well-being measure. However, this would only be the case if the PANAVA was included after the questions regarding distance-learning. In our study, the PANAS scale was included only after child demographics were asked, thus reducing the possibility of priming effects.

As to the reasons why parents are having such difficulties with distance-learning, we can speculate that there might be a few variables at play that have been appeared in other studies in Germany. As we had mentioned before, German parents are not even remotely acquainted with homeschooling practices, while at the same time teachers feel ill-prepared when facing the prospect of distance-teaching (Eickelmann et al., 2019). The lack of appropriate infrastructure for digital-teaching in Germany (Bos et al., 2014; Eickelmann et al., 2019; Huber et al., 2020) might also have helped to widen the gap between parents and teachers regarding what can be expected from each other in order to help students adapt to distance-learning. This lack of coordination among parents and teachers might have consequences for students if not resolved, and some might even prove to be long-term.

We had expected that being the main “caregiver” would have an impact on well-being and household dynamic, nevertheless this was not the case. However, longitudinal studies such as the ones performed by Huebener et al. (2020) and Möhring et al. (2021), found that relative well-being (previous years versus during the pandemic) has had a significant downward trend for mothers. It must be noted that, in these studies, (subjective) well-being was measured as satisfaction with different aspects such as general life satisfaction, family satisfaction and satisfaction with childcare for the former study; and family life satisfaction and work satisfaction for the latter. Possible reasons for this discrepancy may lie in the different operationalization of and instruments to measure well-being.

Although most of the variance in the PANAS scales was unaccounted for in our model, still these stressors (with the exception of “caregiver”) did have a significant impact on parental well-being and household dynamics, and therefore, might contribute to the build-up or curbing of family/parental stress. Most of the participants in our sample seemed to be doing quite well, despite the circumstances. Nevertheless, the lockdown measures have been extended and societies have had to live under these circumstances for over a year now; the outlook might have changed during that time and the amount of accumulated stress could be considerable. Now that vaccination against COVID-19 has been started in many countries, families will have to slowly re-adapt to their previous *status quo*. It remains to be seen if a “return to normality” is attainable, yet setbacks caused by sporadic outbreaks may make it a protracted process. Forcing parents to constantly re-adapt to changing circumstances might prove to be another stressor, which they must face. Therefore, we consider longitudinal or experience sampling studies of families with school-aged children best suited to understand the many effects the COVID-19 pandemic has had and will have on them.

Several implications can be drawn from our study. First, it is not the mitigating measures *per se* that are related to parents’ well-being (with the exceptions of teaching assistance), but rather the household dynamics. Governments should set or keep up their social support structures for families, offering assistance, and open spaces for parents and children. Second, parents differ with regard to how much they can and want to take on the role as “assistant teacher,” and schools differ regarding the expectations they have for parents’ support and how explicit they made their expectations. Previous research (Fan and Chen, 2001; Sheldon and Epstein, 2005; Fan and Williams, 2010; Jeynes, 2012) has shown that parent-school-cooperation can improve students’ educational outcomes, but that its potentials are not fully exhausted in Germany (Wild, 2003; Sacher et al., 2019). Our study did show that distance-learning may not only affect students but also their parents: we found that taking on the role as “assistant teacher” was an important factor related to parents’ well-being. Hence, school should aim at improving partnerships, setting up clear expectations, and assisting parents who have fewer means to support their children.

Third, due to the school closures, German schools were forced to set up technical structures and implement tools for distant teaching and learning. Now in place, schools will and should keep using these tools. Communicating with parents about which support they need regarding novel technical tools, and how technology can be supportive instead of an additional burden seems therefore warranted.

### Limitations

Not included in our analysis are many other factors that could impact families and have been commonplace during the pandemic: job loss, loss of income, separation, and death of loved ones as more direct factors but also psychological aspects such as pressure, lack of control (e.g., Miller et al., 2020), or more specific parenting scales (e.g., Brown et al., 2020; Ren et al., 2020). Due to questionnaire-length concerns, we focused on our main points of interest; namely, we were especially interested in how the school closure, and with it, the new role of parents as assistant teachers, influenced parents’ well-being and the household dynamics. Due to the novelty of the situation, this aspect is still understudied.

It must be highlighted that the sample used for our study was not a representative sample because of the convenience sampling procedure. Self-selection bias is to be expected. Additionally, our measures of well-being and household dynamics rely on self-report about events before and after the COVID-19 pandemic, which might be biased due to memory and/or other variables. Therefore, it is important to juxtapose the findings in this paper with longitudinal studies on parental well-being that happened to occur just before the pandemic started, as they have the advantage of contemporaneous well-being assessments. Moreover, such a study would also serve as a quasi-experimental study that could more convincingly prove a causal relationship among variables.

Similar to the majority of studies conducted during the first lockdown our sample was preponderantly female (Brose et al., 2020; Porsch and Porsch, 2020; Sander et al., 2020; Wildemann and Hosenfeld, 2020; Blume et al., 2021; Steinmayr et al., 2021). Women are usually overrepresented in psychological studies (Dickinson et al., 2012), but in the case of the present study the reason most likely lies in the instruction. As previously mentioned, respondents were required to be the main caregiver of the child (or children), which usually is the mother in Germany. Another limitation is that only one questionnaire was filled in per household, therefore, we lack the information how the partner (in the cases where there was one) perceived the situation. As aforementioned, results from previous studies showed that fathers’ and mothers’ well-being was differentially affected by the pandemic and its mitigating measures (Möhring et al., 2021). However, when considering the important role that the main caregiver has for the family system, and hence, for the well-being and development of the children, we decided to focus on this individual to obtain a report on the entire family.

Moreover, we considered an online questionnaire as the best method under the lockdown circumstances. As other researchers (e.g., Heller and Zuügel, 2020; Huber and Helm, 2020; Lorenz et al., 2020), we exclusively used online media to recruit our sample, such as Facebook, Twitter, or e-mail. However, this of course biased the sample to those families with internet access and those more familiar with internet surveys, who tend to be more educated and of a higher socioeconomic class. This is also reflected in our sample which was skewed with regard to socioeconomic and educational background. Hence, it can be assumed that these families had less difficulties assisting their children with schoolwork and faced fewer socioeconomic problems during the pandemic (and were therefore more available to answer our questionnaire). Consequently, our results may not be able to be extrapolated to participants who do not fit in these categories. Other studies, especially conducted during the first wave and working with convenience samples, face this problem as well (cp. Steinmayr et al., 2021). Future studies should aim to include more diverse sample or a more representative sample, for example by using different methodological approaches (e.g., focus group interviews or representative sampling); as findings with such samples would provide a more complete picture of how German families are coping with the pandemic-induced lifestyle changes, which can then be extrapolated to countries with similar conditions.

Finally, another limitation is the correlational nature of our study. This implies that we cannot prove causation among the studied variables, only correlations.

## Conclusion

For our German convenience sample, we observed that during the early COVID-19 pandemic parental well-being in general was quite positive despite parents’ struggle with their new role as “assistant teachers.” Parents of school-aged children have mostly been able to establish positive dynamics in their households given the extra time they get to spend with their children, and this has in turn benefited their well-being. However, it is important to bridge the gap between parents and teachers regarding distance-learning because it is a source of stress for parents and a prolonged period under these circumstances may lead to a breakdown in parent-child and parent-teacher relationships and negative long-term consequences for the students.

## Data Availability Statement

The raw data supporting the conclusions of this article will be made available by the authors, without undue reservation.

## Ethics Statement

Ethical review and approval was not required for the current study in accordance with the local legislation and institutional requirements. All ethical guidelines provided by the German Psychological Society (DGPs) were followed. The patients/participants provided their written informed consent to participate in this study.

## Author Contributions

AH contributed to the conception and design of the study. DC-R implemented the survey, performed the statistical analysis, and was the lead author of the manuscript with guidance and contributions from AH. AH and DC-R organized the database. Both authors contributed to manuscript revision, read, and approved the submitted version.

## Conflict of Interest

The authors declare that the research was conducted in the absence of any commercial or financial relationships that could be construed as a potential conflict of interest.

## Publisher’s Note

All claims expressed in this article are solely those of the authors and do not necessarily represent those of their affiliated organizations, or those of the publisher, the editors and the reviewers. Any product that may be evaluated in this article, or claim that may be made by its manufacturer, is not guaranteed or endorsed by the publisher.

## Footnotes

1 Naturally, schools differed on how smoothly and efficaciously they implemented the change and in their expectations regarding the supporting role of the parents.

2 This term (alternative spelling “home-office”) will be used throughout this paper to shorten “working from home.” However, it should be noted that this is more of a German term, different from the American English term “home office,” which refers to an office space at home, or the British English “Home Office,” which refers to a governmental department (German Language Blog, 2020). In Germany, another term that is used in official documents is “telework.”

3 Our questionnaire was in German and was aimed (and disseminated) mainly to German residents. However, we did not discard participants from other German-speaking countries from the final sample (such as Austria or Switzerland), which took similar mitigation measures roughly during the same timeframe (Schweizer Bundesrat, 2020; Blum and Dobrotic, 2021).

4
www.soscisurvey.de

5 School-closures were ongoing as this study was finished.

6 Note that this correlation table cannot be used to reproduce the subsequent model, since in our model we do not use subscale scores, but the data on each individual item. The correlation table on which our model is based on can be found in Supplementary Appendix B.
