# Supplementary material for: Juggling School and Work From Home: Results From a Survey on German Families With School-Aged Children During the Early COVID-19 Lockdown
Source: Front Psychol. 2022 Jan 31;12:734257. doi: 10.3389/fpsyg.2021.734257 (PMC8841713; doi:10.3389/fpsyg.2021.734257)
Supplement: Supplementary file 1 [file Data_Sheet_1.pdf]

Appendix A

Questionnaire Items
Item ID. Original German (English translation)

CD01. In welchem Jahr wurde Ihr Kind geboren? Bitte auswählen
(In what year was your child born? Please choose)
Antworten: 2005-2014, Andere
(Answer options: 2005-2014, Other)
CD03. Ihr Kind ist...
(Your child is...)
Antworten: Männlich, Weiblich, Divers
(Answer options: Male, Female, Diverse.)
CD04. In welche Schule geht Ihr Kind?
(What school type does your child attend?)
Antworten: Berufschule, Gemeinschaftschule/Gesamtschule, Grundschule, Gymnasium, Hauptschule/Werkrealschule, Integrierte Sekundarschule (integrierte Haupt- und Realschulen), Realschulen, Schule mit Förderschwerpunkt/sonderpädagogisches Bildungs- und Beratungszentrum, Sonstige Schule (Waldorf, Montessori, u.a.).
CD05. In welche Klassenstufe geht Ihr Kind?
(What grade is your child in?)
Antworten: 1-13, Anders
(Answer options: 1st-13th, Other)
PN01. Wie haben Sie sich in den letzten Tagen gefühlt?
(How have you felt in the last days?)
Antworten: Likertskala von 1 „gar nicht“ bis 5 „äußerst“
(Answer options: Likert scale from 1 „not at all“ to 5 „extremely“)
PN01_01. aktiv (active)
PN01_02. bekümmert (distressed)
PN01_03. verärgert (upset)
PN01_04. erschrocken (startled)
PN01_05. feindselig (hostile)
PN01_06. angeregt (inspired)
PN01_07. gereizt (irritable)
PN01_08. begeistert (enthusiastic)
PN01_09. nervös (nervous)
PN01_10. entschlossen (determined)
PN01_11. aufmerksam (attentive)
PN01_12. ängstlich (scared)
PN01_13. verunsichert (unsettled)
RO03. Wenn Sie an die Schulschließung denken, wie sehr stimmen Sie den folgenden Aussagen zu?
(When you think about the school closures, how much do you agree with the following statements?)
RO03_01. Mein Kind reagiert gereizt, wenn ich ihm Schulaufgaben erkläre.
(My child reacts annoyed when I explain schoolwork to him/her)
Antworten: Likertskala von 1 „stimme überhaupt nicht zu“ bis 4 „stimme voll und ganz zu“
(Answer options: Likert scale from 1“strongly disagree” to 4 “strongly agree”)
RO04. Im Folgen finden Sie einige Situationen und Aktivitäten: Bitte vergleichen Sie, ob Sie die Situationen und Aktivitäten seit der Schulschließung häufiger oder seltener mit Ihrem Kind erlebt haben als in der Zeit vor der Schulschließung.

(In the following you will find some situations and activities: Please compare if these activities or situations have become more or less common with your child since the school closures compared to before the school closures)
Antworten: Likertskala von 1 „sehr viel seltener“ bis 5 „sehr viel häufiger“ und „gilt nicht“
(Answer options: Likert scale from 1 “much less often” to 5 “much more often”)
RO04_01. Gemeinsames kochen/essen
(cooking/eating together)
RO04_02. gemeinsame Freizeitaktivitäten (Sport, Musizieren, handwerkliche und künstlerische Beschäftigung, Spielen)
(doing recreational activities together [sports, playing music, arts and crafts, playing])
RO04_03. gemeinsames Fernsehen
(watching TV together)
RO04_04. Streit um die Erledigung der Schulaufgaben
(dispute about homework)
RO04_05. Streit um die Überprüfung der Schulaufgaben
(dispute when proofreading homework)
RO04_06. Streit um die Nutzung von Smartphone, Tablett o.ä.
(disputes about the usage of smartphones, tablets, or similar)
RO04_07. Streit über andere Themen
(dispute about other topics)
RO04_08. Längere Unterhaltung über ein Thema
(long talks about a topic)
RO04_09. Streit mit den Geschwistern
(disputes with siblings)
PD02. Sind Sie selbst Lehrerin oder Lehrer?
(Are you a teacher?)
Antworten: Ja, Nein.
(Answer options: Yes, No)
PD04. Welchen höchsten Bildungsabschluss haben Sie?
(What is your maximum education level?)
Antworten: Keine, Volkschule/Hauptschule, Realschulabschluss/Mittlere Reife, Hochschulreife/Abitur, Hochschulabschluss.
(Answer options: None, elementary education, intermediary education, higher secondary education, university degree)
PD21. Wohnen Sie in Konstanz?
(Do you live in Konstanz?)
Antworten: Ja, Nein
(Answer options: Yes, No)
PD18. In welchem Bundesland wohnen Sie?
(In what state do you live?)
Antworten: [16 Bundesländer]
(Answer options: the 16 German federal states)
PD06. Wie stehen Sie zum Kind?
(What is your relationship to the child?)
Antworten: die Mutter/Stiefmutter/Pflegemutter, der Vater/Stiefvater/Pflegevater, sonstige Person (z. B. Großmutter/-vater)
(Answer options: Mother/Stepmother/Foster mother, Father/Stepfather/Foster father, Other Person [for example, Grandmother/-father])
PD07. Sind Sie alleinerziehend?
(Are you a single parent?)
Antworten: Ja, teilweise oder überwiegend, nein.
(Answer options: Yes, partly/mostly, no)

PD08. Wer ist bei Ihnen zuhause normalerweise die Hauptbetreuerin oder der Hauptbetreuer für das Kind?
(In your home, who is the main caregiver of the child?)
Antworten: Ich selbst, mein/e Partner/in, wir beide zusammen, sonstige Person(en).
(Answer options: Myself, my partner, both my partner and I together, Other person[s])
PD09. In welchem Erwerbs- bzw. Tätigkeitsverhältnis stehen Sie?
(What is your employment status?)
Antworten: in Rente, Arbeitssuchen/nichterwerbstätig, in Erziehungs-urlaub, in Ausbildung/Weiterbildung/Studium, Erwerbstätig mit Minijob, Angestellt erwerbstätig in Teilzeit, Angestellt erwerbstätig in Vollzeit, Selbstständig erwerbstätig.
(Answer options: Retired, looking for a job/unemployed, in parental leave, student, mini-job, employed part-time, employed full-time, self-employed/freelance)
PD10. Können oder müssen Sie derzeit (bspw. aufgrund von Corona-Schutzmaßnahmen) von zuhause aus arbeiten (Homeoffice)?
(Can you or must you currently work from home due to the corona mitigation measures?)
Antworten: Ja, teilweise, nein.
(Answer options: Yes, partly, no)
PD16. Wie beurteilen Sie Ihre gegenwärtige finanzielle Situation? Mit unserem derzeitigen Einkommen, können wir als Familie...
(How do you judge your current financial situation? With our income, we can…)
Antworten: bequem leben, zurechtkommen, nur schwer zurechtkommen, nur sehr schwer zurechtkommen.
(Answer options: live comfortably, get by, get by with difficulties, barely get by)
