# Supplementary material for: Juggling School and Work From Home: Results From a Survey on German Families With School-Aged Children During the Early COVID-19 Lockdown
Source: Front Psychol. 2022 Jan 31;12:734257. doi: 10.3389/fpsyg.2021.734257 (PMC8841713; doi:10.3389/fpsyg.2021.734257)
Supplement: Supplementary file 2 [file Table_1.pdf]

Appendix B

Table 7.

Zero-order correlations for PANAS and HHD scale items, caregiver, homeoffice and teaching assistance variables.

Variable	1	2	3	4	5	6	7	8	9	10	11	12	13	14	15	16	17	18	19	20	21	22	23	24	25
1) PANAS_P1	–																								
2) PANAS_P6	.35 ***	–																							
3) PANAS_P8	.27 ***	.41 ***	–																						
4) PANAS_P10	.36 ***	.41 ***	.38 ***	–																					
5) PANAS_P11	.31 ***	.29 ***	.31 ***	.52 ***	–																				
6) PANAS_N2	–.08 **	–.09 **	–.23 ***	–.15 ***	–.11 ***	–																			
7) PANAS_N3	–.08 **	–.07 *	–.18 ***	–.12 ***	–.16 ***	.46 ***	–																		
8) PANAS_N4	–.08 *	.01	–.16 ***	–.02	–.01	.47 ***	.29 ***	–																	
9) PANAS_N5	–.06 ***	.02	–.11 ***	–.07 *	–.12 ***	.36 ***	.57 ***	.30 ***	–																
10) PANAS_N7	–.09 ***	–.06 *	–.20 ***	–.14 ***	–.18 ***	.47 ***	.63 ***	.23 ***	.50 ***	–															
11) PANAS_N9	–.10 ***	–.03	–.20 ***	–.15 ***	–.09 ***	.51 ***	.36 ***	.49 ***	.33 ***	.48 ***	–														
12) PANAS_N12	–.09 ***	–.03	–.16 ***	–.12 ***	–.06 *	.50 ***	.23 ***	.53 ***	.24 ***	.31 ***	.57 ***	–													
13) PANAS_N13	–.15 ***	–.04	–.23 ***	–.16 ***	–.08 **	.48 ***	.24 ***	.52 ***	.24 ***	.33 ***	.55 ***	.72 ***	–												
14) HHD_P1	.07 *	.08 **	.09 ***	.07 *	.06 *	–.04	–.06 *	–.02	–.03	–.01	.00	–.03	–.03	–											
15) HHD_P2	.07	.11 ***	.18 ***	.10 ***	.05	–.09 **	–.16 ***	–.09 **	–.08 **	–.12 ***	–.08 **	–.05	–.05	.45 ***	–										
16) HHD_P3	.00	.03	.02	–.03	.01	.02	–.05	.02	–.01	.01	.04	.01	.03	.25 ***	.23 ***	–									
17) HHD_P8	.05 ***	.11 ***	.14 ***	.12 ***	.08 **	–.05	–.12 ***	.02	–.06 *	–.06 *	.01	.03	.02	.28 ***	.33 ***	.17 ***	–								
18) HHD_N4	–.12 ***	–.09 **	–.20 ***	–.16 ***	–.18 ***	.23 ***	.37 ***	.12 ***	.22 ***	.40 ***	.22 ***	.12 ***	.13 ***	–.06 *	–.15 ***	–.03	–.06	–							
19) HHD_N5	–.11 ***	–.10 ***	–.20 ***	–.15 ***	–.11 ***	.20 ***	.29 ***	.11 ***	.17 ***	.34 ***	.20 ***	.14 ***	.12 ***	–.07 *	–.16 ***	–.02	–.06 *	.77 ***	–						
20) HHD_N6	–.10 ***	–.04	–.18 ***	–.10 ***	–.09 **	.14 ***	.22 ***	.11 ***	.13 ***	.26 ***	.20 ***	.12 ***	.16 ***	–.04	–.11 ***	.10 **	.00	.42 ***	.41 ***	–					
21) HHD_N7	–.06 *	–.06 *	–.16 ***	–.12 ***	–.11 ***	.19 ***	.30 ***	.09 **	.20 ***	.37 ***	.21 ***	.13 ***	.14 ***	–.10 ***	–.13 ***	–.03	–.03	.37 ***	.36 ***	.35 ***	–				
22) HHD_N9	–.09 **	–.06	–.13 ***	–.09 **	–.06	.19 ***	.30 ***	.09 **	.22 ***	.36 ***	.15 ***	.11 ***	.17 ***	–.01	–.08 *	–.02	–.01	.31 ***	.29 ***	.32 ***	.51 ***	–			
23) HHD_N6_2	–.12 ***	–.08 **	–.20 ***	–.16 ***	–.19 ***	.22 ***	.31 ***	.11 ***	.18 ***	.35 ***	.22 ***	.16 ***	.18 ***	–.02	–.08 **	.02	–.05	.50 ***	.47 ***	.31 ***	.26 ***	.20 ***	–		
24) Caregiver	.01	–.02	.00	.00	–.02	–.05	–.01	–.01	–.04	–.02	.02	.03	.02	–.03	–.03	.02	-.03	-.00	–.02	.04 *	.00	-.01	.00	–	
25) Homeoffice	.08 **	.07 *	.01	.03	–.03	–.03	–.04	–.04	.00	.07	.04	.00	.00	.18 ***	.16 ***	.09 **	.09 **	.04	.00	.00	–.04	–.04	–.02	–.07 *	–
26) Teaching assistance	.03	.01	–.02	–.01	–.02	.15	.22 ***	.07 ***	.12 **	.19 ***	.08 **	.08 **	.08 **	.03	.02	–.01	.03	.21 ***	.16 ***	.13 ***	.15 ***	.15 ***	.20 ***	.02	.03

Note: PANAS _P refers to items of the positive PANAS subscale, PANAS_N indicates the negative subscale. Same principle for HHD item names. The number at the end of the item label indicates the sequence in which items were presented to participants, except in the case for item HHD_N6, which was presented in a separate section between the PANAS and HHD scales * $p < .05$, ** $p < .01$, *** $p < .001$
